# Negative interferences by calcium dobesilate in the detection of five serum analytes involving Trinder reaction-based assays

**DOI:** 10.1371/journal.pone.0192440

**Published:** 2018-02-12

**Authors:** Xiuzhi Guo, Li’an Hou, Yicong Yin, Jie Wu, Fang Zhao, Liangyu Xia, Xinqi Cheng, Qian Liu, Li Liu, Ermu Xu, Ling Qiu

**Affiliations:** Department of Laboratory Medicine, Peking Union Medical College Hospital, Chinese Academic Medical Science and Peking Union Medical College, Beijing, P. R. China; Universidad de Guadalajara, MEXICO

## Abstract

Previously, we reported the strong negative interference of calcium dobesilate, a vasoprotective agent, in creatinine assays involving the Trinder reaction. It is hypothesized that a similar effect occurs in the detection of uric acid (UA), total cholesterol (TC), triglycerides (TG), high-density lipoprotein cholesterol (HDL-C), and low-density lipoprotein cholesterol (LDL-C). The interferences of calcium dobesilate during the detection of the five serum analytes were investigated on automated systems/analysers, and the effects were compared among eight different assay systems for each analyte. A calcium dobesilate standard was added into two sets of the blank serum pools of each analyte at final concentrations of 0, 2, 4, 8, 16, 32, and 64 μg/mL. The percentage deviation of each analyte value was calculated between each drug concentration and the drug-free samples. The clinically acceptable error levels for UA, TC, TG, HDL-C, and LDL-C were defined as ±4.87%, ±4.1%, ±9.57%, ±5.61%, and ±5.46%, respectively. The observed interference was concentration dependent for each analyte. In the presence of 16 μg/mL calcium dobesilate, which was within the therapeutic range, all seven Trinder reaction-based UA assay systems, two TG assay systems, two HDL-C assay systems and one TC assay system exhibited negative drug interferences. Calcium dobesilate negatively interferes with the detection of UA, TG, TC, and HDL-C in assay systems based on the Trinder reaction. The effect was most significant in UA and TG detection.

## Introduction

The Trinder reaction is a fundamental process employed in a variety of clinical biochemical tests. Currently, Trinder-coupled chromogenic reactions are used for the determination of creatinine (Cr), uric acid (UA), cholesterol, and triglycerides (TG). Originally, phenol was used as the chromogenic agent for the Trinder reaction. With the application of a number of alternative chromogenic materials, such as 2,4-dichlorophenol, the sensitivity of this method has been improved significantly. However, due to the poor substrate specificity of catalytic peroxidase for the Trinder reaction, multiple substances, such as ascorbic acid [1,2], etamsylate [3], N-acetylcysteine [4], and bilirubin [5], can interfere with the assay’s results. Recently, we confirmed that the medication calcium dobesilate produces significant negative interferences with the determination of creatinine using sarcosine oxidase-based assays, and the extent of interference differs significantly among the different assay systems [6].

The influence of drugs on the results of clinical testing is a common but often overlooked problem. As described in detail previously [6], calcium dobesilate (calcium 2,5-dihydroxybenzenesulfonate) is a vasoprotectant [7] that is widely used to treat diabetic retinopathy [8], chronic venous insufficiency [9], and various microangiopathy [10]. Recent studies have revealed its protective effects on diabetic nephropathy [11] and gentamicin-induced acute kidney injury [12] and have suggested potential protective effects against intestinal ischaemia-reperfusion injury by increasing antioxidant capacity [13]. Orally administered calcium dobesilate is present in the body as the parent drug following absorption and it is excreted through the kidney and the intestines [14]. According to the pharmacokinetic data, calcium dobesilate reaches a peak plasma concentration of 8 μg/mL at 6 h following a single oral dosage of 500 mg [14–15]. Currently, the recommended clinical dose for calcium dobesilate is 500 mg three times per day, and its steady-state plasma concentration is estimated to be above 15 μg/mL [14].

In the previous study [6], we proposed the consumption of hydrogen peroxide (H_2_O_2_) produced in the Trinder reaction by calcium dobesilate as the potential mechanism of its interference with creatinine detection by sarcosine oxidase-based assays. It can be inferred that calcium dobesilate may interfere with other assays based on the same Trinder reaction. Therefore, we examined commercially available assay systems utilizing the Trinder reaction for the quantification of the following analytes: uric acid, total cholesterol (TC), triglycerides, high-density lipoprotein cholesterol (HDL-C), and low-density lipoprotein cholesterol (LDL-C). For each analyte, more than ten imported and domestic assay kits were surveyed for the content in the specifications and assay protocol. It was found that only the Roche product specifies the issue of interference by calcium dobesilate for UA and TG. With rapid advancements in the *in vitro* diagnostic industry, it is important to clarify whether calcium dobesilate interferes with the analysis of other serum analytes and, if so, the extent to which it interferes in different assays. To answer these questions, we evaluated the interference of calcium dobesilate in different assay systems for the detection of UA, TC, TG, HDL-C, and LDL-C using an *in vitro* addition method.

## Materials and methods

### Base serum for interference experiments

A panel of two base serum pools for each analyte were used to evaluate the level of calcium dobesilate interference. Concentrations for the low and high serum pools were based on recommendations from the Clinical and Laboratory Standards Institute (CLSI) EP7-A2 guidelines [16], which for UA were 200 and 500 μmol/L, for TC were 3.88 mmol/L and 6.47 mmol/L, for TG were 1.7 mmol/L and 5.6 mmol/L, for HDL-C were 0.9 mmol/L and 1.8 mmol/L, respectively. For LDL-C, there is no recommended concentration, so we used the same serum pools we used for HDL-C. The serum was prepared by the full mixing of nonicteric and nonhaemolysed samples obtained from patients receiving physical examinations or hospitalized at Peking Union Medical College Hospital (PUMCH) in September 2015. Approximately ten to twelve serum samples that were close to the concentration mentioned above were selected from the LIS system for each analyte. The patient medical histories were viewed to ensure that these patients were free of calcium dobesilate treatment. The study protocol was approved by the ethics committee of PUMCH (S-732). Written informed consent was given by all patients.

### Reagents and equipment

Eight commercially available UA, TC, TG, HDL-C, and LDL-C assay kits were evaluated using the following manufacturer/analyser combinations routinely used in hospitals as shown in Table 1. All assay kits were based on the Trinder reaction except for the Siemens UA assay kit, which uses the UA-UV method. The calcium dobesilate standard (SML-0488, ≥98%) used in the *in vitro* experiments was purchased from Sigma Chemicals (St. Louis, MO).

**Table 1:** Reagents and equipment.

analyte	manufacturer/analyser							
UA	Roche^a^/ Cobas c702	Beckman^b^/ Beckman AU5800	Beckman/ Beckman DXC600i	Siemens^d^*/ RxL Max	Ortho/Vitros^e^ / Vitros 250	Maker^f^/ Beckman AU5800	Leadman^g^/ Hitachi 7180	Biosino^h^/ Hitachi 7180
TG, TC, HDL-C, LDL-C	Roche/ Cobas c702	Beckman/ Beckman AU5800	Sekisui^c^/ Beckman AU5800	Siemens/ RxL Max	Ortho/Vitros / Vitros 250	Maker/ Beckman AU5800	Leadman/ Hitachi 7180	Biosino/ Hitachi 7180

1.) Abbreviations

a.) Roche, Hoffmann-La Roche Ltd., Basel, Switzerland

b.) Beckman, Beckman Coulter, Inc., Brea, CA

c.) Sekisui, Sekisui Chemical Co., Ltd., Hitachi, Tokyo, Japan

d.)Siemens, Siemens Healthcare Diagnostics, Inc., Newark, DE, US

e.) Ortho/Vitros, Ortho Clinical Diagnostics, Johnson & Johnson, Rochester, NY

f.) Maker, Sichuan Maker Biotechnology Co., Ltd., Chengdu, China

g.) Leadman, Leadman Biochemistry Co., Ltd., Beijing, China; Biosino, Bio-Technology and Science Inc., Beijing, China

h.) Biosino, Bio-Technology and Science Inc., Beijing, China

2.) *Siemens UA assay kit uses a UA-UV method, and other assay kits were all based on the Trinder reaction.

### *In vitro* interference experiments

A calcium dobesilate standard was added to the base serum pools to prepare the dose-response series using the sequential mixing method described in the CLSI EP7-A2 guidelines [16]. The final calcium dobesilate concentrations for each series were 0, 2, 4, 8, 16, 32, and 64 μg/mL. The specimens were dispensed of and placed into insulated containers maintained at 2–8°C for delivery to testing locations. The UA, TC, TG, HDL-C, LDL-C levels were measured using the eight commercial assay kits within 4 hours. Specimens were analysed in triplicate within one analytical run to obtain an average value. An internal quality control was applied during the experiments to ensure test quality. Based on the biological variations, the acceptable limits of deviations for the UA, TC, TG, HDL-C, and LDL-C was ±4.87%, ±4.1%, ±9.57%, ±5.61% and ±5.46%, respectively [17].

### Statistical analysis

The Graphpad prism version 5.0 for Windows (GraphPad Software, SanDiego, CA) was used for data analysis and graphing. The percentage deviations (y) were calculated with respect to the drug-free specimen and were plotted vs. the calcium dobesilate concentrations (x). The short-dashed horizontal lines indicate the acceptable limits of deviations for each analyte.

## Results

**Fig 1:**
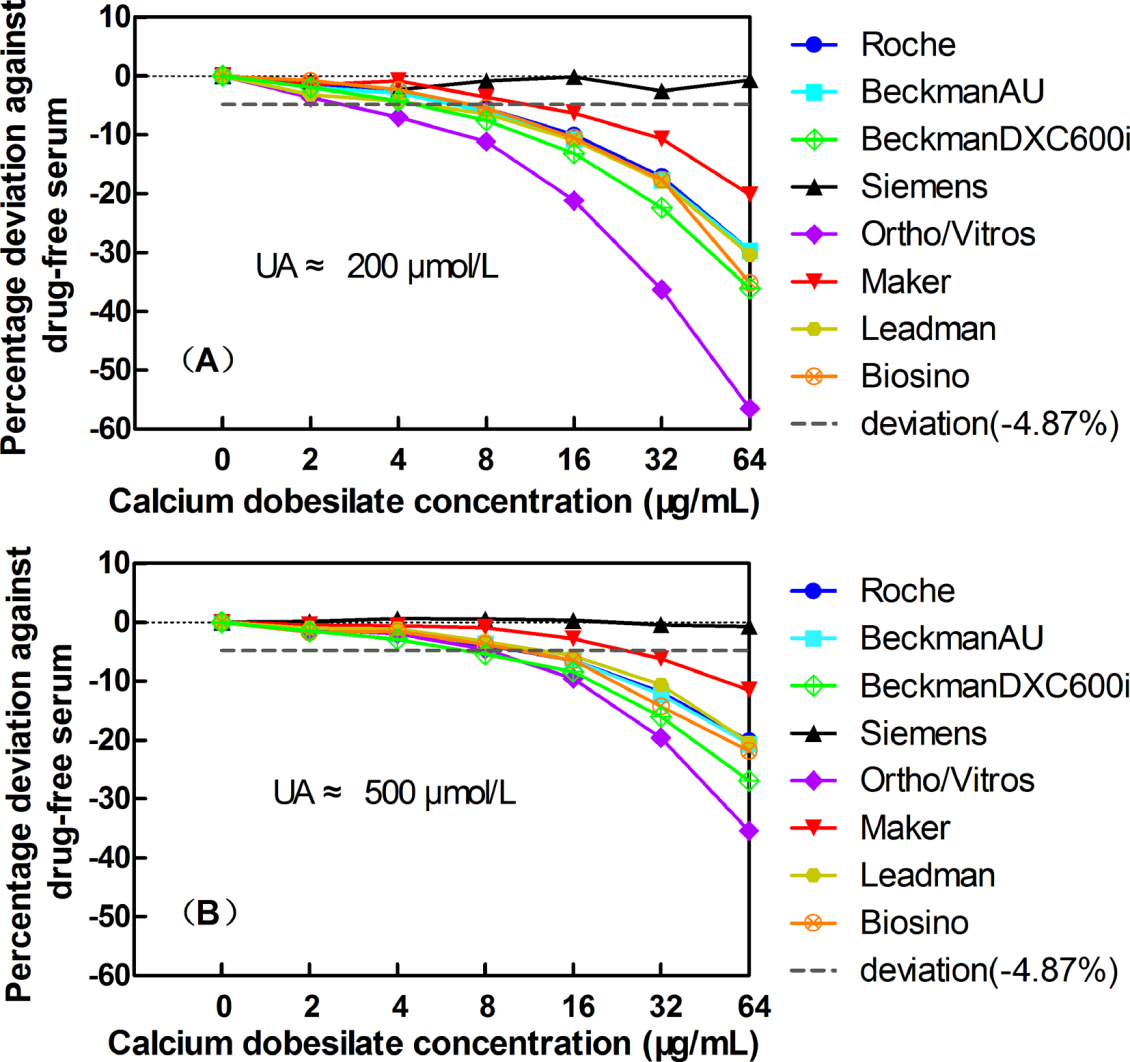
The effects of the exogenous addition of calcium dobesilate on UA quantification in 7 Trinder reaction-based assay systems (Roche, BeckmanAU, BeckmanDXC600i, Siemens, Ortho/Vitros, Maker, Leadman, and Biosino) and one UA-UV method assay (Siemens). Panels A and B show the data for basal UA concentrations of 200 and 500 μmol/L, respectively. The short-dashed horizontal lines indicate the acceptable limits of deviation at -4.87% for UA.

Considering the clinically acceptable deviation of ±4.87% for UA, the exogenous addition of calcium dobesilate clearly exhibited dose-dependent negative interference with the determination of UA in all seven Trinder reaction-based assays (Fig 1). In the presence of 16 μg/mL calcium dobesilate, all seven Trinder reaction-based UA assays exhibited deviations ranging from -6.3% to -21.2% in the low UA serum group (Fig 1A). Furthermore, six of the seven Trinder reaction-based UA assays (except for the Maker assay), exceeded the acceptable limits of deviations for the UA (-4.87%) in the high UA serum group, with deviations ranging from -5.7% to -9.6% (Fig 1B). The same calcium dobesilate concentrations produced significantly different levels of interference among the different assays, with the Ortho/Vitros assay exhibiting the maximum interference and the Maker assay exhibiting the minimum interference. As a control assay, the Siemens system using the UA-UV method did not show any interference (Fig 1).

**Fig 2:**
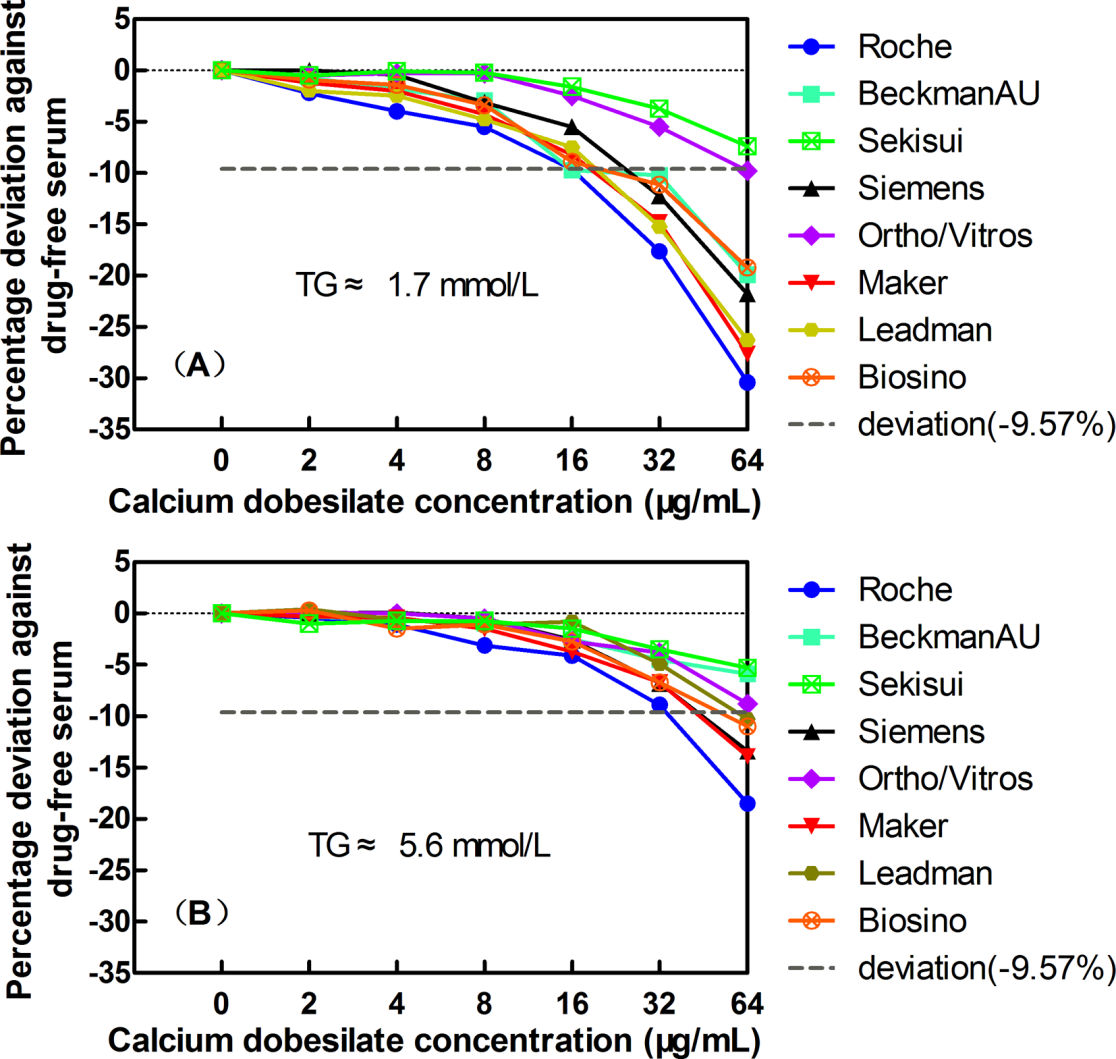
The effects of the exogenous addition of calcium dobesilate on TG quantification in 8 Trinder reaction-based assay systems: Roche, Beckman, Siemens, Ortho/Vitros, Maker, Leadman, and Biosino, Sekisui. Panels A and B show the data for basal TG concentrations of 1.7 mmol/L and 5.6 mmol/L, respectively. The short-dashed horizontal lines indicate the acceptable limits of deviation at -9.57% for TG.

As shown in Fig 2, considering the clinically acceptable deviations of ±9.57% for TG, in the presence of 16 μg/mL calcium dobesilate, only two assay systems (Roche, Beckman AU) exhibited slight negative interference in the low TG serum group, with deviations of -9.6% and -9.7%, respectively (Fig 2). However, as the drug concentration increased, the interference became more noticeable. At calcium dobesilate concentrations of 64 μg/mL, seven TG assays (Roche, Beckman, Siemens, Ortho/Vitros, Maker, Leadman, and Biosino) in the low TG serum group and five TG assays (Roche, Siemens, Maker, Leadman, and Biosino) in the high TG serum group exceeded the acceptable limits of deviations for the TG (-9.57%). The Sekisui assay system displayed relatively strong anti-interference performance and showed deviations of -7.4% and -5.3% at calcium dobesilate concentrations of 64 μg/mL in the low and high TG serum groups, respectively.

**Fig 3:**
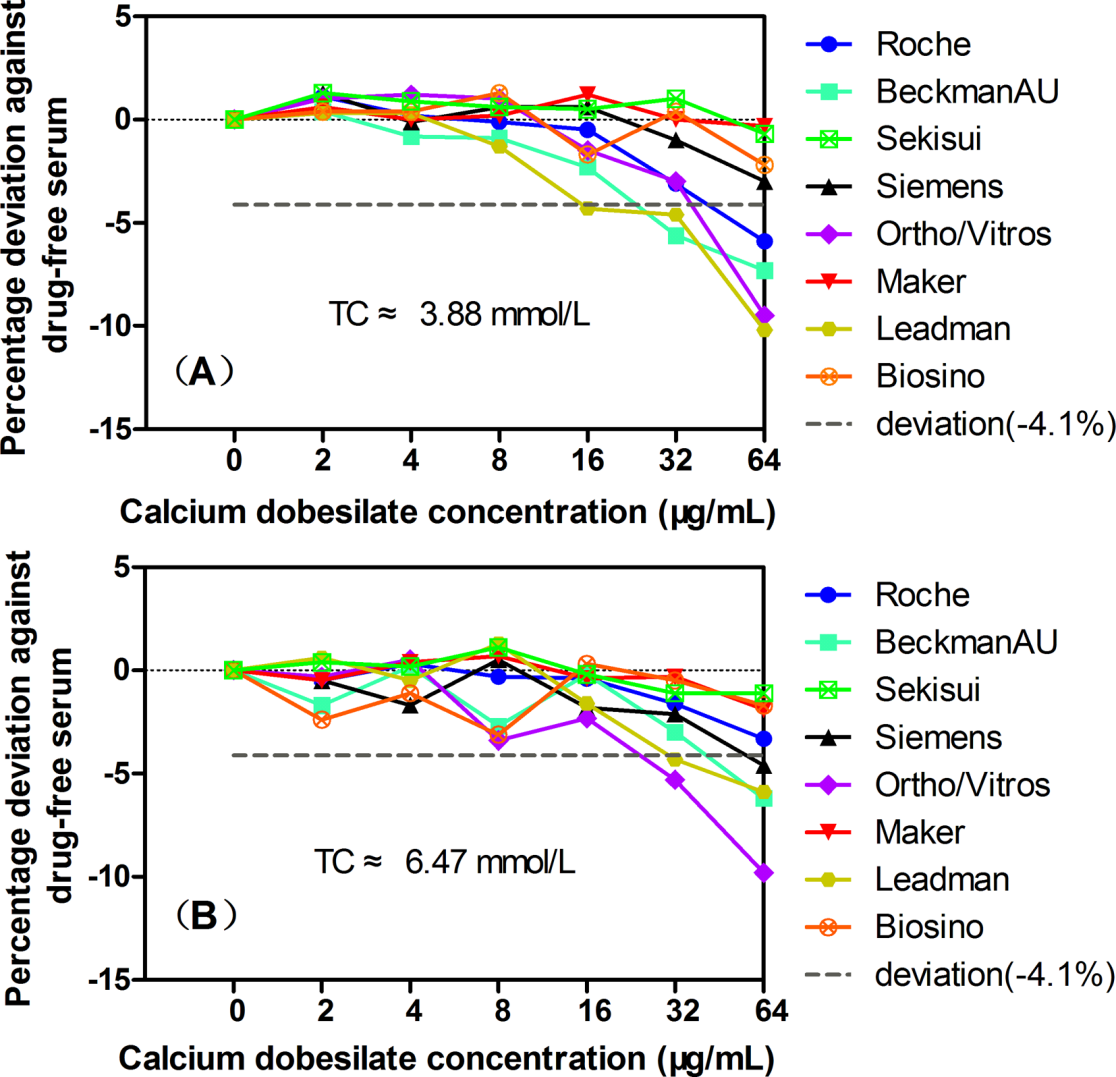
The effects of the exogenous addition of calcium dobesilate on the TC quantification in 8 Trinder reaction-based assay systems: Roche, Beckman, Siemens, Ortho/Vitros, Maker, Leadman, and Biosino, Sekisui. Panels A and B show the data for basal TC concentrations of 3.88 mmol/L and 6.47 mmol/L, respectively. The short dashed horizontal lines indicate the acceptable limits of deviation at -4.1% for TC.

As shown in Fig 3, considering the clinically acceptable deviations of ±4.1% for TC, when the exogenous calcium dobesilate concentrations were ≤64 μg/mL, no interference was observed for the TC assay systems from Sekisui, Maker, and Biosino in the low and high TC serum groups. Only small negative interferences of -5.9% were observed for the Roche assay in the low TC serum group and -4.6% for the Siemens assay in the high TC serum group at a calcium dobesilate concentration of 64 μg/mL, respectively. In contrast, the Leadman assay system showed a higher interference of -4.3% to -10.2% at calcium dobesilate concentrations of 16 μg/mL and above in the low and high TC serum groups, while the interference for the Beckman AU and Ortho/Vitros systems ranged from -5.6% to -9.8% at calcium dobesilate concentrations of 32 μg/mL and above.

**Fig 4:**
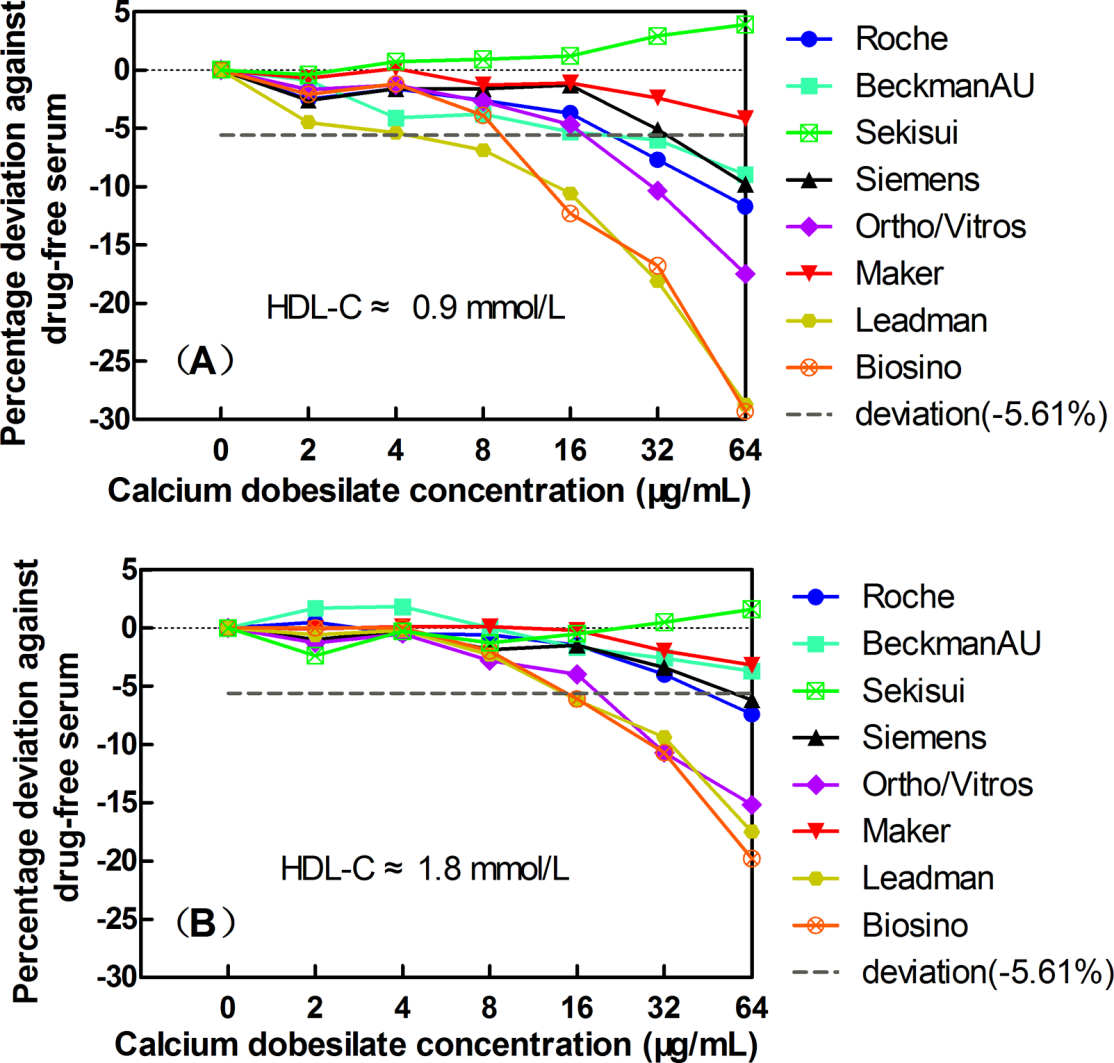
The effects of the exogenous addition of calcium dobesilate on HDL-C quantification in 8 Trinder reaction-based assay systems: Roche, Beckman, Siemens, Ortho/Vitros, Maker, Leadman, and Biosino, Sekisui. Panels A and B show the data for basal HDL-C concentrations of 0.9 mmol/L and 1.8 mmol/L, respectively. The short dashed horizontal lines indicate the acceptable limits of deviation at -5.61% for HDL-C.

The interference in the HDL-C assays was displayed in Fig 4. Considering the clinically acceptable deviations of ±5.61% for HDL-C, when the exogenous calcium dobesilate concentrations were ≤64 μg/mL, no interference was observed for the HDL-C assay systems from Sekisui and Maker in the low and high HDL-C serum groups. At a calcium dobesilate concentration of 16 μg/mL, negative interferences of -10.6%, and -12.3% were observed in the Leadman, and Biosino assays in the low HDL-C serum group (Fig 4A), while the interference in the high HDL-C serum group was -6.1% and -6.2%, respectively (Fig 4B).

**Fig 5:**
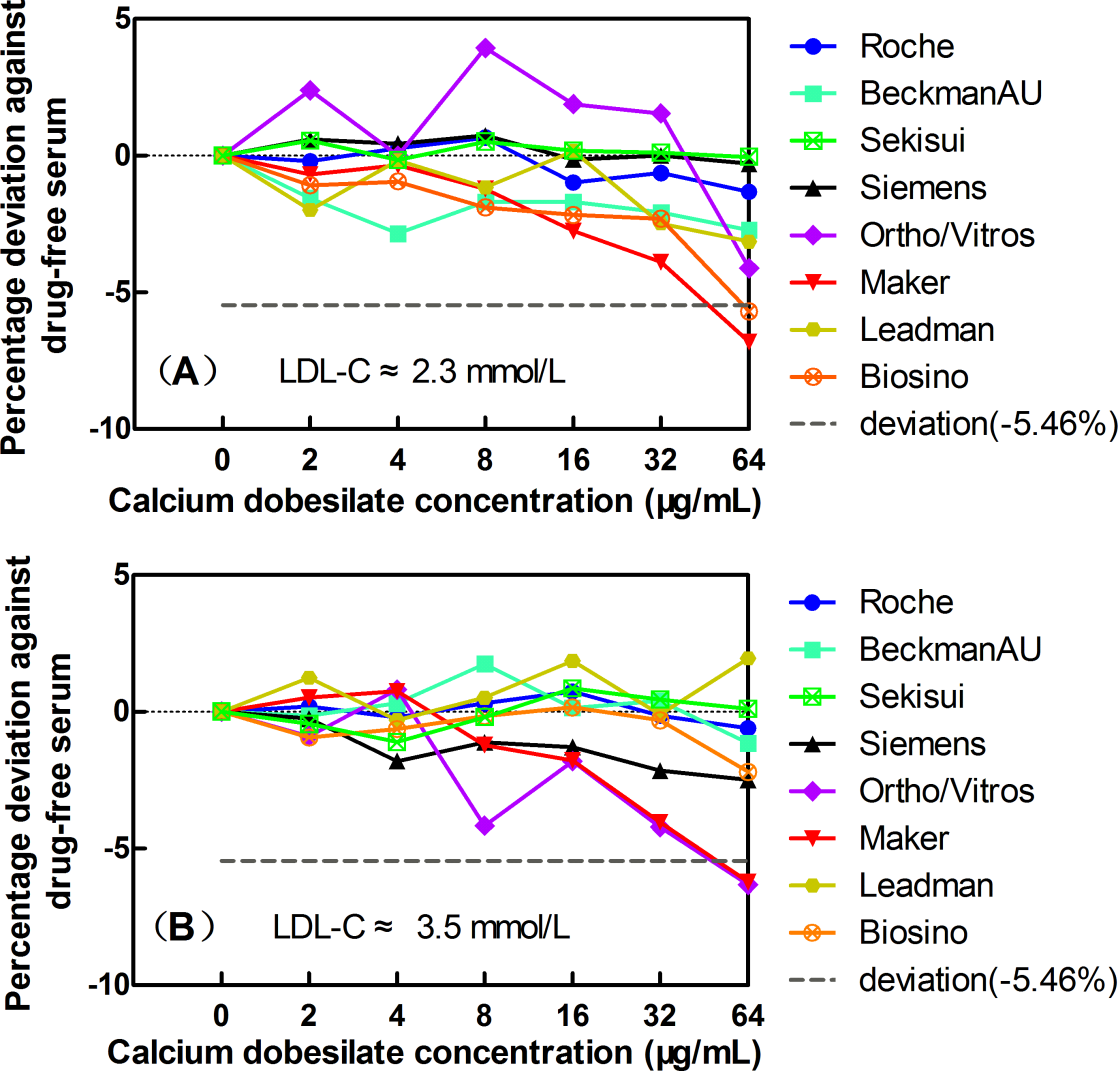
The effects of the exogenous addition of calcium dobesilate on LDL-C quantification in 8 Trinder reaction-based assay systems: Roche, Beckman, Siemens, Ortho/Vitros, Maker, Leadman, and Biosino, Sekisui. Panels A and B show the data for basal HDL-C concentrations of 2.3 mmol/L and 3.5 mmol/L, respectively. The dashed horizontal lines indicate the acceptable limits of deviations at -5.46% for LDL-C.

Considering the clinically acceptable deviations of ±-5.46% as a standard for LDL-C, when the calcium dobesilate concentrations reached 32μg/mL and below, no significant interference was observed for any of the eight LDL-C assay systems (Fig 5). At a calcium dobesilate concentration of 64 μg/mL, only slight negative interferences of -6.8% and -5.7% were observed for the Roche and Biosino assay in the low LDL-C serum group, respectively, while interferences of -6.2% and -6.3% were observed for the Roche and Siemens assays in the high TC serum group, respectively.

## Discussion

This study investigated the interferences of calcium dobesilate in the detection of the five serum analytes. The observed degree of interference is related to the drug concentration in the serum. In our previous study [6], calcium dobesilate was administered to ten volunteers for three days, and the trough and peak serum concentrations were found to be 2.66–8.33 μg/mL and 12.83–23.15 μg/mL, respectively. This study reveals that at a plasma concentration as low as 4 μg/mL, calcium dobesilate can negatively interfere with the measurement of UA in the Ortho/Vitros assay. At a concentration of 16 μg/mL, calcium dobesilate negatively interferes in seven assays for UA, two assays for TG, two assays for HDL-C, and one assay for TC. Importantly, these effects were observed to be dose-dependent; the higher the dosage, the higher the interference. Therefore, the administration of calcium dobesilate in patients will result in sufficiently high serum concentrations of calcium dobesilate to artificially lower the values of the serum analytes, and the interference will be greater if blood samples are collected shortly after a dose. Because calcium dobesilate is primarily excreted through the kidneys, drug accumulation can occur in patients with impaired kidney function, resulting in even more severe interference in the testing results. It has been reported previously that the calcium dobesilate concentration in patients taking calcium dobesilate can be as high as 63.35 μg/mL [6]. The interference is expected to be more substantial at such concentration levels.

As described in our previous study [6], although it has been known for nearly 30 years that calcium dobesilate interferes with creatinine determination using enzymatic methods [18], the mechanism of the interference has not been elucidated in great detail. We hypothesized that the hydroquinone ring in calcium dobesilate can consume the hydrogen peroxide (H_2_O_2_) that serves as the indicator species in the peroxidase reaction, thus producing negative interference. In our study, the Siemens assay kit using the UA-UV method for the detection of UA showed no interference by calcium dobesilate. In this method, the first reaction step is identical to that of the other assay kits. It involves the conversion of uric acid (which absorbs UV light at 293 nm) by uricase to allantoin (which is nonabsorbing at 293 nm), CO_2_ and H_2_O_2_. However, in the second step, the Siemens assay measures the uric acid level by quantifying its concentration based on changes in the absorbance at 293 nm due to the disappearance of uric acid, while other assays rely on the quantification of H_2_O_2_ in the Trinder reaction. The mechanistic differences, along with the results of the interference study, support our hypothesis that the key issue of calcium dobesilate interference is the coloration step in the Trinder reaction.

Nonetheless, questions still remain as to why the extent of interference varies so significantly for different analytes using the same Trinder reaction. Previous studies have shown severe interference in creatinine assays. In this study, interference in the UA and TG tests was significant, while only slight interference was seen in the LDL-C test. Our initial analysis suggests that these results are related to differences in the structure and sensitivity of the chromogen, which could alter the amount of H_2_O_2_ produced in the reaction system. A detailed mechanism of interference needs to be determined to provide solutions to the issue.

The acceptability criteria for interference is very important in evaluating interference. As described in EP7-A2-2005 guidelines [16], the degree of allowable error caused by interference obviously depends on the medical use of the test results. Interference criteria can based on physioloyical variability, derived from clinical experience, or based on analytical variability. In this study, we set the acceptable limits of the deviations for interference base on the bias derived from biologic variation [17]. A different acceptability criteria will lead to different conclusions. The imprecision in the measurements is another important issue in the evaluation of interference, which could change the strength of our conclusions. Therefore, we repeated the measurement of each analytes in serum pools triplicately, and showed mean values in results to reduce the impact of random errors. The detailed imprecision data was shown in the S1–S5 Tables.

In this study, the observed interference was found to be concentration dependent for each analyte. The EP7-A2 guidelines [16] suggest that interference should be initially evaluated at two medical decision concentrations of the analyte. In our results, the observed interferences were greater at a lower basal concentration of analytes (specifically UA, TG and TC). We think the first reason may be because the interferences were compared in terms of their percentage deviation. When the deviation is similar, the lower the value, the greater the percentage deviation. Another reason may be that drugs in the reaction system can consume a fixed amount of hydrogen peroxide. We therefore speculate that the reaction system with higher basal concentrations of analytes produces more hydrogen peroxide and produces fewer disturbances.

According to statistics from a previous study [6], 13,599 patients received calcium dobesilate treatment at Beijing Union Medical College Hospital in 2014, highlighting the need for special concern and attention to the interference issue. Calcium dobesilate is primarily prescribed to treat and prevent diabetic retinopathy, and diabetes patients often have complications of hyperuricaemia and dyslipidaemia. Negative interference of the medication with UA and TG testing can cause inaccurate estimation of the UA and lipid levels in patients. Previously, the interference of calcium dobesilate in the enzymatic creatinine assays was observed in our laboratory because creatinine levels are relatively stable. Therefore, any short-term fluctuations are easily noticeable to physicians and lab personnel. However, the levels of UA, TC, TG, and HDL-C are substantially affected by diet, and variations in testing results can be easily overlooked. If the laboratory has more than one detection system for these analytes, the differences between results of systems is conducive to confirm the existence of interference.

It is pertinent to raise the awareness of such interference so that clinical physicians can make prudent judgements on the levels of creatinine, uric acid, and lipids in patients under calcium dobesilate therapy. If possible, the clinician can recommend that patients temporarily stop taking drugs 3 to 5 days before the biochemical examination. If this is not possible, it is recommended to complete the blood draw before taking the drugs in order to minimize the degree of interference. If necessary, LC-MS / MS methods can be used to determine Cr, UA, TC, TG and HDL-C concentrations without interferences in patients who are taking calcium dobesilate. For UA, the Siemens UV method assay can also be used as an alternative method.

This study is limited to the *in vitro* addition method, and the *in vivo* interference in volunteers and patients was not evaluated. However, we have completed the *in vivo* experiments. Unlike creatinine, these five analytes were found to be greatly affected by the diet. The results fluctuated in participants *in vivo* experiments, and this cannot be used to draw conclusions about their own changes or changes caused by drug interference. However, based on previous creatinine studies, the *in vitro* results agreed quite well with the *in vivo* results and can be used to assess the interferences *in vivo*. This is related to the fact that calcium dobesilate is present in the body as the parent drug.

## Conclusions

In this study, we confirmed through *in vitro* addition experiments that calcium dobesilate negatively interferes with the detection of UA, TC, TG, and HDL-C in assay systems based on the Trinder reaction. The effect is most significant in UA and TG and varies among assay systems from different suppliers. Consequently, physicians should practise prudent judgement regarding the reported levels of creatinine, UA, TC, TG, and HDL-C in patients taking calcium dobesilate.

## Supporting information

S1 Table The mean (μmol/L) and coefficient of variation (CV) for UA triplicately measured in 8 systems. (DOC)

S2 Table The mean (mmol/L) and coefficient of variation (CV) for TG triplicately measured in 8 systems. (DOCX)

S3 Table The mean (mmol/L) and coefficient of variation (CV) for TC triplicately measured in 8 systems. (DOCX)

S4 Table The mean (mmol/L) and coefficient of variation (CV) for HDL-C triplicately measured in 8 systems. (DOCX)

S5 Table The mean (mmol/L) and coefficient of variation (CV) for LDL-C triplicately measured in 8 systems. (DOCX)
