# Supplementary material for: Negative interferences by calcium dobesilate in the detection of five serum analytes involving Trinder reaction-based assays
Source: PLoS One. 2018 Feb 12;13(2):e0192440. doi: 10.1371/journal.pone.0192440 (PMC5809042; doi:10.1371/journal.pone.0192440)
Supplement: S1 Table — (DOC) [file pone.0192440.s001.doc]

**S1 Table. The mean (μmol/L) and coefficient of variation (CV) for UA triplicately measured in 8 systems.**

| calcium dobesilate concentrations | | Roche | |  | Beckman | |  | Siemens | |  | Ortho/Vitros | |  | Maker | |  | Leadman | |  | Biosino | |  | Beckman DXC600i | |
| --- | --- | --- | --- | --- | --- | --- | --- | --- | --- | --- | --- | --- | --- | --- | --- | --- | --- | --- | --- | --- | --- | --- | --- | --- |
|  |  | mean | CV |  | mean | CV |  | mean | CV |  | mean | CV |  | mean | CV |  | mean | CV |  | mean | CV |  | mean | CV |
| low UA serum group | 0 | 195.73 | 0.38 |  | 205.54 | 0.20 |  | 192.67 | 1.97 |  | 207.53 | 0.37 |  | 193.70 | 1.15 |  | 200.33 | 2.46 |  | 201.99 | 0.38 |  | 206.67 | 0.28 |
|  | 2 | 192.80 | 0.71 |  | 201.73 | 0.29 |  | 191.00 | 1.39 |  | 199.80 | 0.58 |  | 190.80 | 0.26 |  | 194.00 | 0.89 |  | 200.42 | 0.61 |  | 202.67 | 0.57 |
|  | 4 | 190.17 | 0.45 |  | 199.50 | 0.33 |  | 188.00 | 1.41 |  | 193.07 | 0.26 |  | 192.03 | 0.91 |  | 191.67 | 0.30 |  | 197.09 | 0.71 |  | 198.00 | 0.51 |
|  | 8 | 184.97 | 0.54 |  | 192.97 | 0.60 |  | 191.00 | 1.05 |  | 184.27 | 0.08 |  | 186.67 | 0.99 |  | 187.33 | 0.31 |  | 191.14 | 0.87 |  | 191.00 | 0.00 |
|  | 16 | 176.13 | 0.43 |  | 183.60 | 0.22 |  | 192.33 | 1.20 |  | 163.53 | 0.89 |  | 181.53 | 0.71 |  | 178.33 | 0.32 |  | 180.73 | 0.25 |  | 179.33 | 0.32 |
|  | 32 | 162.30 | 0.59 |  | 168.93 | 0.12 |  | 187.67 | 1.54 |  | 132.13 | 0.39 |  | 173.07 | 1.54 |  | 164.33 | 0.35 |  | 166.44 | 0.98 |  | 160.33 | 0.36 |
|  | 64 | 137.63 | 0.29 |  | 144.43 | 0.28 |  | 191.33 | 1.09 |  | 90.27 | 0.65 |  | 154.67 | 1.26 |  | 139.33 | 0.41 |  | 130.95 | 0.96 |  | 132.00 | 0.76 |
| high UA serum group | 0 | 478.97 | 0.36 |  | 493.83 | 0.12 |  | 469.00 | 0.64 |  | 483.70 | 0.34 |  | 468.20 | 0.41 |  | 469.67 | 0.12 |  | 486.74 | 0.33 |  | 476.33 | 0.32 |
|  | 2 | 472.40 | 0.04 |  | 489.27 | 0.08 |  | 469.67 | 0.44 |  | 477.67 | 0.22 |  | 465.63 | 0.17 |  | 465.00 | 0.74 |  | 478.54 | 0.76 |  | 469.00 | 0.00 |
|  | 4 | 470.30 | 0.30 |  | 484.37 | 0.39 |  | 471.67 | 0.53 |  | 474.27 | 0.42 |  | 465.50 | 0.15 |  | 464.67 | 0.76 |  | 478.71 | 0.29 |  | 462.33 | 0.54 |
|  | 8 | 460.50 | 0.39 |  | 476.03 | 0.50 |  | 471.33 | 0.24 |  | 461.43 | 0.34 |  | 464.20 | 0.96 |  | 454.33 | 0.25 |  | 468.44 | 0.30 |  | 450.67 | 0.64 |
|  | 16 | 448.20 | 0.82 |  | 461.93 | 0.05 |  | 470.33 | 0.32 |  | 437.03 | 1.11 |  | 454.87 | 0.98 |  | 443.00 | 0.78 |  | 455.28 | 0.05 |  | 436.33 | 0.26 |
|  | 32 | 422.17 | 0.78 |  | 433.10 | 0.21 |  | 467.33 | 1.58 |  | 388.83 | 0.34 |  | 439.17 | 1.25 |  | 419.67 | 0.14 |  | 417.09 | 0.06 |  | 400.33 | 0.38 |
|  | 64 | 382.53 | 0.57 |  | 391.70 | 0.33 |  | 465.67 | 0.45 |  | 312.53 | 0.23 |  | 414.23 | 1.06 |  | 374.00 | 0.80 |  | 380.56 | 0.09 |  | 348.00 | 0.29 |
