# Supplementary material for: Negative interferences by calcium dobesilate in the detection of five serum analytes involving Trinder reaction-based assays
Source: PLoS One. 2018 Feb 12;13(2):e0192440. doi: 10.1371/journal.pone.0192440 (PMC5809042; doi:10.1371/journal.pone.0192440)
Supplement: S2 Table — (DOCX) [file pone.0192440.s002.docx]

**S2 Table. The mean (mmol/L) and coefficient of variation (CV) for TG triplicately measured in 8 systems.**

| calcium dobesilate concentrations | | Roche | |  | Beckman | |  | Siemens | |  | Ortho/Vitros | |  | Maker | |  | Leadman | |  | Biosino | |  | Sekisui | |
| --- | --- | --- | --- | --- | --- | --- | --- | --- | --- | --- | --- | --- | --- | --- | --- | --- | --- | --- | --- | --- | --- | --- | --- | --- |
|  |  | mean | CV |  | mean | CV |  | mean | CV |  | mean | CV |  | mean | CV |  | mean | CV |  | mean | CV |  | mean | CV |
| low TG serum group | 0 | 1.73 | 0.09 |  | 1.83 | 0.84 |  | 1.70 | 0.34 |  | 2.02 | 0.29 |  | 1.87 | 0.51 |  | 1.86 | 0.31 |  | 1.84 | 0.54 |  | 1.70 | 1.49 |
|  | 2 | 1.69 | 0.42 |  | 1.81 | 0.32 |  | 1.70 | 0.34 |  | 2.01 | 0.29 |  | 1.85 | 0.26 |  | 1.83 | 0.84 |  | 1.82 | 0.32 |  | 1.69 | 1.31 |
|  | 4 | 1.66 | 0.18 |  | 1.79 | 0.32 |  | 1.69 | 1.02 |  | 2.01 | 0.00 |  | 1.83 | 0.94 |  | 1.82 | 1.39 |  | 1.81 | 0.64 |  | 1.69 | 2.29 |
|  | 8 | 1.63 | 0.62 |  | 1.77 | 0.33 |  | 1.64 | 0.93 |  | 2.01 | 0.50 |  | 1.79 | 0.71 |  | 1.77 | 0.65 |  | 1.78 | 0.86 |  | 1.69 | 0.27 |
|  | 16 | 1.56 | 0.13 |  | 1.65 | 0.86 |  | 1.60 | 1.30 |  | 1.97 | 0.29 |  | 1.72 | 0.29 |  | 1.72 | 1.21 |  | 1.68 | 0.89 |  | 1.67 | 0.49 |
|  | 32 | 1.43 | 0.52 |  | 1.64 | 0.61 |  | 1.49 | 1.34 |  | 1.91 | 0.61 |  | 1.60 | 0.39 |  | 1.58 | 1.67 |  | 1.64 | 0.93 |  | 1.63 | 0.94 |
|  | 64 | 1.20 | 0.94 |  | 1.46 | 0.79 |  | 1.33 | 0.87 |  | 1.82 | 0.00 |  | 1.35 | 0.85 |  | 1.37 | 0.42 |  | 1.49 | 0.39 |  | 1.57 | 0.40 |
| high TG serum group | 0 | 4.80 | 0.81 |  | 5.16 | 0.89 |  | 4.94 | 0.94 |  | 5.32 | 0.33 |  | 5.42 | 0.18 |  | 5.32 | 1.93 |  | 5.20 | 0.40 |  | 5.16 | 0.68 |
|  | 2 | 4.78 | 0.69 |  | 5.14 | 0.40 |  | 4.94 | 0.47 |  | 5.32 | 0.39 |  | 5.41 | 0.35 |  | 5.35 | 2.00 |  | 5.21 | 0.69 |  | 5.11 | 0.31 |
|  | 4 | 4.75 | 0.26 |  | 5.13 | 0.70 |  | 4.94 | 0.54 |  | 5.32 | 0.33 |  | 5.40 | 0.46 |  | 5.29 | 1.11 |  | 5.12 | 0.63 |  | 5.12 | 0.38 |
|  | 8 | 4.65 | 0.55 |  | 5.11 | 0.30 |  | 4.91 | 0.42 |  | 5.29 | 0.44 |  | 5.34 | 0.33 |  | 5.27 | 0.29 |  | 5.14 | 0.55 |  | 5.12 | 0.73 |
|  | 16 | 4.60 | 0.18 |  | 5.03 | 0.60 |  | 4.81 | 0.43 |  | 5.18 | 0.56 |  | 5.22 | 0.28 |  | 5.28 | 0.66 |  | 5.06 | 1.01 |  | 5.09 | 0.49 |
|  | 32 | 4.37 | 0.42 |  | 4.93 | 0.42 |  | 4.60 | 0.55 |  | 5.12 | 0.34 |  | 5.06 | 0.12 |  | 5.06 | 0.71 |  | 4.85 | 0.52 |  | 4.98 | 0.96 |
|  | 64 | 3.91 | 0.16 |  | 4.86 | 0.66 |  | 4.27 | 0.27 |  | 4.85 | 0.31 |  | 4.67 | 1.41 |  | 4.77 | 0.67 |  | 4.62 | 0.33 |  | 4.89 | 1.13 |
