# Supplementary material for: Negative interferences by calcium dobesilate in the detection of five serum analytes involving Trinder reaction-based assays
Source: PLoS One. 2018 Feb 12;13(2):e0192440. doi: 10.1371/journal.pone.0192440 (PMC5809042; doi:10.1371/journal.pone.0192440)
Supplement: S3 Table — (DOCX) [file pone.0192440.s003.docx]

**S3 Table. The mean (mmol/L) and coefficient of variation (CV) for TC triplicately measured in 8 systems.**

| calcium dobesilate concentrations | | Roche | |  | Beckman | |  | Siemens | |  | Ortho/Vitros | |  | Maker | |  | Leadman | |  | Biosino | |  | Sekisui | |
| --- | --- | --- | --- | --- | --- | --- | --- | --- | --- | --- | --- | --- | --- | --- | --- | --- | --- | --- | --- | --- | --- | --- | --- | --- |
|  |  | mean | CV |  | mean | CV |  | mean | CV |  | mean | CV |  | mean | CV |  | mean | CV |  | mean | CV |  | mean | CV |
| low TC serum group | 0 | 3.82 | 0.47 |  | 3.93 | 1.92 |  | 3.83 | 0.66 |  | 3.75 | 1.41 |  | 3.89 | 0.74 |  | 3.71 | 0.98 |  | 3.72 | 1.78 |  | 3.73 | 1.52 |
|  | 2 | 3.86 | 0.34 |  | 3.94 | 2.21 |  | 3.88 | 0.54 |  | 3.79 | 1.59 |  | 3.92 | 1.15 |  | 3.72 | 0.78 |  | 3.74 | 0.41 |  | 3.78 | 0.37 |
|  | 4 | 3.82 | 0.77 |  | 3.89 | 1.80 |  | 3.82 | 0.60 |  | 3.79 | 1.00 |  | 3.89 | 0.78 |  | 3.72 | 0.67 |  | 3.74 | 1.08 |  | 3.77 | 0.58 |
|  | 8 | 3.81 | 1.25 |  | 3.89 | 1.69 |  | 3.85 | 0.94 |  | 3.79 | 1.50 |  | 3.90 | 1.43 |  | 3.66 | 0.58 |  | 3.77 | 0.27 |  | 3.76 | 0.37 |
|  | 16 | 3.80 | 0.71 |  | 3.84 | 1.89 |  | 3.85 | 0.69 |  | 3.69 | 1.03 |  | 3.94 | 0.25 |  | 3.55 | 0.67 |  | 3.66 | 2.77 |  | 3.75 | 0.83 |
|  | 32 | 3.70 | 0.27 |  | 3.71 | 1.02 |  | 3.79 | 0.26 |  | 3.64 | 0.97 |  | 3.89 | 0.59 |  | 3.54 | 0.64 |  | 3.74 | 1.21 |  | 3.77 | 0.87 |
|  | 64 | 3.59 | 1.13 |  | 3.64 | 1.79 |  | 3.71 | 0.95 |  | 3.39 | 1.55 |  | 3.88 | 1.12 |  | 3.33 | 0.78 |  | 3.64 | 0.55 |  | 3.71 | 0.57 |
| high TC serum group | 0 | 6.41 | 0.27 |  | 6.56 | 0.44 |  | 6.52 | 0.53 |  | 6.18 | 1.48 |  | 6.62 | 0.71 |  | 6.32 | 0.78 |  | 6.40 | 0.96 |  | 6.29 | 0.49 |
|  | 2 | 6.37 | 1.38 |  | 6.45 | 0.45 |  | 6.49 | 0.47 |  | 6.16 | 1.91 |  | 6.59 | 0.70 |  | 6.36 | 0.67 |  | 6.24 | 1.21 |  | 6.32 | 1.02 |
|  | 4 | 6.42 | 0.31 |  | 6.57 | 0.87 |  | 6.41 | 0.95 |  | 6.21 | 0.34 |  | 6.65 | 0.23 |  | 6.29 | 1.02 |  | 6.33 | 1.03 |  | 6.30 | 0.32 |
|  | 8 | 6.39 | 0.28 |  | 6.39 | 1.49 |  | 6.55 | 0.85 |  | 5.97 | 1.31 |  | 6.67 | 0.95 |  | 6.40 | 0.87 |  | 6.20 | 2.30 |  | 6.36 | 0.31 |
|  | 16 | 6.38 | 0.33 |  | 6.55 | 0.70 |  | 6.40 | 0.47 |  | 6.04 | 0.44 |  | 6.60 | 0.89 |  | 6.22 | 0.62 |  | 6.42 | 0.50 |  | 6.28 | 0.33 |
|  | 32 | 6.30 | 0.02 |  | 6.36 | 1.92 |  | 6.38 | 0.90 |  | 5.85 | 0.86 |  | 6.60 | 0.23 |  | 6.05 | 0.89 |  | 6.36 | 1.05 |  | 6.22 | 1.10 |
|  | 64 | 6.19 | 0.67 |  | 6.16 | 0.66 |  | 6.22 | 0.43 |  | 5.58 | 1.26 |  | 6.50 | 0.62 |  | 5.95 | 0.92 |  | 6.29 | 0.00 |  | 6.22 | 1.89 |
