# Supplementary material for: Negative interferences by calcium dobesilate in the detection of five serum analytes involving Trinder reaction-based assays
Source: PLoS One. 2018 Feb 12;13(2):e0192440. doi: 10.1371/journal.pone.0192440 (PMC5809042; doi:10.1371/journal.pone.0192440)
Supplement: S4 Table — (DOCX) [file pone.0192440.s004.docx]

**S4 Table. The mean (mmol/L) and coefficient of variation (CV) for HDL-C triplicately measured in 8 systems.**

| calcium dobesilate concentrations | | Roche | |  | Beckman | |  | Siemens | |  | Ortho/Vitros | |  | Maker | |  | Leadman | |  | Biosino | |  | Sekisui | |
| --- | --- | --- | --- | --- | --- | --- | --- | --- | --- | --- | --- | --- | --- | --- | --- | --- | --- | --- | --- | --- | --- | --- | --- | --- |
|  |  | mean | CV |  | mean | CV |  | mean | CV |  | mean | CV |  | mean | CV |  | mean | CV |  | mean | CV |  | mean | CV |
| low HDL-C serum group | 0 | 1.04 | 1.10 |  | 1.06 | 3.27 |  | 1.02 | 0.57 |  | 0.99 | 1.01 |  | 0.92 | 1.35 |  | 1.10 | 0.52 |  | 1.11 | 1.87 |  | 1.01 | 1.34 |
|  | 2 | 1.02 | 0.88 |  | 1.05 | 0.95 |  | 0.99 | 1.01 |  | 0.97 | 1.19 |  | 0.92 | 0.27 |  | 1.05 | 0.55 |  | 1.09 | 1.83 |  | 1.00 | 0.36 |
|  | 4 | 1.03 | 0.30 |  | 1.02 | 2.84 |  | 1.00 | 1.73 |  | 0.98 | 0.59 |  | 0.92 | 1.58 |  | 1.04 | 2.00 |  | 1.10 | 0.91 |  | 1.01 | 0.50 |
|  | 8 | 1.02 | 0.99 |  | 1.02 | 2.59 |  | 1.00 | 1.00 |  | 0.96 | 0.60 |  | 0.91 | 1.39 |  | 1.03 | 0.56 |  | 1.07 | 1.62 |  | 1.01 | 1.96 |
|  | 16 | 1.01 | 0.43 |  | 1.00 | 3.04 |  | 1.00 | 1.52 |  | 0.94 | 0.61 |  | 0.91 | 0.93 |  | 0.99 | 0.59 |  | 0.98 | 3.26 |  | 1.02 | 1.47 |
|  | 32 | 0.96 | 1.30 |  | 1.00 | 3.00 |  | 0.97 | 0.73 |  | 0.89 | 0.65 |  | 0.90 | 0.48 |  | 0.90 | 1.69 |  | 0.93 | 1.25 |  | 1.04 | 0.64 |
|  | 64 | 0.92 | 0.57 |  | 0.97 | 2.20 |  | 0.92 | 0.63 |  | 0.82 | 0.71 |  | 0.89 | 0.46 |  | 0.79 | 0.73 |  | 0.79 | 1.94 |  | 1.06 | 2.85 |
| high HDL-C serum group | 0 | 1.95 | 0.58 |  | 1.82 | 3.67 |  | 1.94 | 0.00 |  | 1.99 | 0.58 |  | 1.77 | 1.22 |  | 1.77 | 1.17 |  | 1.97 | 1.67 |  | 1.86 | 1.11 |
|  | 2 | 1.96 | 0.50 |  | 1.85 | 0.63 |  | 1.92 | 0.90 |  | 1.97 | 1.06 |  | 1.77 | 0.85 |  | 1.76 | 0.33 |  | 1.97 | 1.56 |  | 1.82 | 1.34 |
|  | 4 | 1.94 | 0.34 |  | 1.85 | 0.00 |  | 1.93 | 1.19 |  | 1.98 | 1.54 |  | 1.77 | 0.84 |  | 1.77 | 0.56 |  | 1.97 | 1.43 |  | 1.86 | 0.94 |
|  | 8 | 1.94 | 0.37 |  | 1.82 | 0.64 |  | 1.90 | 0.30 |  | 1.94 | 0.30 |  | 1.77 | 0.95 |  | 1.73 | 0.88 |  | 1.93 | 1.89 |  | 1.84 | 1.46 |
|  | 16 | 1.92 | 0.81 |  | 1.79 | 0.32 |  | 1.91 | 0.52 |  | 1.91 | 0.30 |  | 1.76 | 1.02 |  | 1.66 | 1.51 |  | 1.85 | 1.29 |  | 1.85 | 0.34 |
|  | 32 | 1.87 | 0.65 |  | 1.77 | 0.98 |  | 1.87 | 1.11 |  | 1.78 | 0.56 |  | 1.73 | 0.39 |  | 1.61 | 1.57 |  | 1.76 | 1.33 |  | 1.87 | 0.78 |
|  | 64 | 1.81 | 0.16 |  | 1.75 | 0.99 |  | 1.82 | 0.55 |  | 1.69 | 0.59 |  | 1.71 | 0.50 |  | 1.46 | 0.39 |  | 1.58 | 1.56 |  | 1.89 | 0.42 |
