# Supplementary material for: Negative interferences by calcium dobesilate in the detection of five serum analytes involving Trinder reaction-based assays
Source: PLoS One. 2018 Feb 12;13(2):e0192440. doi: 10.1371/journal.pone.0192440 (PMC5809042; doi:10.1371/journal.pone.0192440)
Supplement: S5 Table — (DOCX) [file pone.0192440.s005.docx]

**S5 Table. The mean (mmol/L) and coefficient of variation (CV) for LDL-C triplicately measured in 8 systems.**

| calcium dobesilate concentrations | | Roche | |  | Beckman | |  | Siemens | |  | Ortho/Vitros | |  | Maker | |  | Leadman | |  | Biosino | |  | Sekisui | |
| --- | --- | --- | --- | --- | --- | --- | --- | --- | --- | --- | --- | --- | --- | --- | --- | --- | --- | --- | --- | --- | --- | --- | --- | --- |
|  |  | mean | CV |  | mean | CV |  | mean | CV |  | mean | CV |  | mean | CV |  | mean | CV |  | mean | CV |  | mean | CV |
| low LDL-C serum group | 0 | 2.22 | 0.70 |  | 2.57 | 2.81 |  | 2.28 | 1.27 |  | 1.95 | 2.64 |  | 2.30 | 0.49 |  | 2.02 | 0.86 |  | 2.46 | 1.47 |  | 2.26 | 0.64 |
|  | 2 | 2.22 | 0.60 |  | 2.53 | 0.68 |  | 2.29 | 0.44 |  | 1.99 | 3.20 |  | 2.28 | 1.22 |  | 1.98 | 1.75 |  | 2.43 | 0.63 |  | 2.27 | 1.16 |
|  | 4 | 2.23 | 0.84 |  | 2.50 | 2.61 |  | 2.29 | 1.01 |  | 1.95 | 3.50 |  | 2.29 | 1.03 |  | 2.02 | 0.29 |  | 2.44 | 1.03 |  | 2.26 | 0.92 |
|  | 8 | 2.24 | 0.13 |  | 2.53 | 0.23 |  | 2.29 | 0.25 |  | 2.02 | 2.90 |  | 2.27 | 0.82 |  | 2.00 | 2.02 |  | 2.41 | 2.43 |  | 2.27 | 0.52 |
|  | 16 | 2.20 | 0.41 |  | 2.53 | 0.91 |  | 2.27 | 0.51 |  | 1.98 | 2.04 |  | 2.23 | 0.81 |  | 2.02 | 0.57 |  | 2.41 | 1.46 |  | 2.26 | 0.98 |
|  | 32 | 2.21 | 0.94 |  | 2.52 | 0.83 |  | 2.28 | 0.67 |  | 1.98 | 2.11 |  | 2.21 | 0.63 |  | 1.97 | 2.64 |  | 2.40 | 1.20 |  | 2.26 | 0.62 |
|  | 64 | 2.19 | 0.28 |  | 2.50 | 1.06 |  | 2.27 | 0.44 |  | 1.87 | 3.20 |  | 2.14 | 0.49 |  | 1.96 | 0.30 |  | 2.32 | 1.29 |  | 2.26 | 0.21 |
| high LDL-C serum group | 0 | 3.95 | 0.13 |  | 4.31 | 0.61 |  | 3.87 | 1.07 |  | 3.32 | 1.83 |  | 3.78 | 0.40 |  | 3.24 | 0.99 |  | 4.22 | 0.76 |  | 3.78 | 0.82 |
|  | 2 | 3.96 | 1.02 |  | 4.30 | 0.94 |  | 3.86 | 0.54 |  | 3.29 | 3.45 |  | 3.80 | 0.62 |  | 3.28 | 0.35 |  | 4.18 | 1.63 |  | 3.76 | 0.84 |
|  | 4 | 3.95 | 1.13 |  | 4.32 | 0.71 |  | 3.80 | 0.55 |  | 3.35 | 1.05 |  | 3.81 | 0.69 |  | 3.23 | 1.53 |  | 4.19 | 0.95 |  | 3.74 | 1.40 |
|  | 8 | 3.97 | 0.32 |  | 4.39 | 3.39 |  | 3.83 | 0.45 |  | 3.17 | 0.67 |  | 3.73 | 0.14 |  | 3.25 | 1.08 |  | 4.21 | 1.26 |  | 3.77 | 0.20 |
|  | 16 | 3.98 | 0.72 |  | 4.32 | 0.94 |  | 3.82 | 0.92 |  | 3.26 | 0.81 |  | 3.71 | 0.53 |  | 3.30 | 1.07 |  | 4.22 | 1.52 |  | 3.81 | 2.26 |
|  | 32 | 3.95 | 0.70 |  | 4.33 | 1.27 |  | 3.79 | 0.46 |  | 3.18 | 1.75 |  | 3.63 | 0.39 |  | 3.23 | 1.99 |  | 4.20 | 1.07 |  | 3.80 | 0.57 |
|  | 64 | 3.93 | 0.16 |  | 4.26 | 2.38 |  | 3.78 | 1.07 |  | 3.11 | 2.25 |  | 3.54 | 0.91 |  | 3.30 | 0.61 |  | 4.12 | 1.75 |  | 3.79 | 0.25 |
